# Ginkgo Biloba Extract Ameliorates Oxidative Phosphorylation Performance and Rescues Aβ-Induced Failure

**DOI:** 10.1371/journal.pone.0012359

**Published:** 2010-08-24

**Authors:** Virginie Rhein, Maria Giese, Ginette Baysang, Fides Meier, Stefania Rao, Kathrin L. Schulz, Matthias Hamburger, Anne Eckert

**Affiliations:** 1 Neurobiology Laboratory for Brain Aging and Mental Health, Psychiatric University Clinics, University of Basel, Basel, Switzerland; 2 Institute of Pharmaceutical Biology, University of Basel, Basel, Switzerland; Case Western Reserve University, United States of America

## Abstract

**Background:**

Energy deficiency and mitochondrial failure have been recognized as a prominent, early event in Alzheimer's disease (AD). Recently, we demonstrated that chronic exposure to amyloid-beta (Aβ) in human neuroblastoma cells over-expressing human wild-type amyloid precursor protein (APP) resulted in (i) activity changes of complexes III and IV of the oxidative phosphorylation system (OXPHOS) and in (ii) a drop of ATP levels which may finally instigate loss of synapses and neuronal cell death in AD. Therefore, the aim of the present study was to investigate whether standardized Ginkgo biloba extract LI 1370 (GBE) is able to rescue Aβ-induced defects in energy metabolism.

**Methodology/Principal Findings:**

We used a high-resolution respiratory protocol to evaluate OXPHOS respiratory capacity under physiological condition in control (stably transfected with the empty vector) and APP cells after treatment with GBE. In addition, oxygen consumption of isolated mitochondria, activities of mitochondrial respiratory enzymes, ATP and reactive oxygen species (ROS) levels as well as mitochondrial membrane mass and mitochondrial DNA content were determined. We observed a general antioxidant effect of GBE leading to an increase of the coupling state of mitochondria as well as energy homeostasis and a reduction of ROS levels in control cells and in APP cells. GBE effect on OXPHOS was even preserved in mitochondria after isolation from treated cells. Moreover, these functional data were paralleled by an up-regulation of mitochondrial DNA. Improvement of the OXPHOS efficiency was stronger in APP cells than in control cells. In APP cells, the GBE-induced amelioration of oxygen consumption most likely arose from the modulation and respective normalization of the Aβ-induced disturbance in the activity of mitochondrial complexes III and IV restoring impaired ATP levels possibly through decreasing Aβ and oxidative stress level.

**Conclusions/Significance:**

Although the underlying molecular mechanisms of the mode of action of GBE remain to be determined, our study clearly highlights the beneficial effect of GBE on the cellular OXPHOS performance and restoration of Aβ-induced mitochondrial dysfunction.

## Introduction

Standardized Ginkgo biloba extract (GBE) derived from dried leaves of Ginkgo tree is a valuable therapeutic drug for the treatment of memory impairment and dementia including Alzheimer's disease (AD). A number of clinical studies repeatedly showed the improvement of cognitive symptoms in the elderly and in AD patients [1]–[5] but negative data have also been published. The discussion about the benefits of GBE for different indications has been revitalized after the publication of two major trials: (i) the study by McCarney and colleagues [6] demonstrating no evidence of effectiveness of GBE in mild to moderate dementia and (ii) the GEM study by DeKosky and colleagues [7] showing no favourable effect of GBE for the prevention of dementia onset in older people without or with only mild cognitive impairment. GBE exhibits a complex mode of action. Notably, multiple effects on mitochondrial function and on the apoptotic pathway that seems to be crucial for its beneficial effects in AD were reported: stabilization of mitochondrial membrane potential, improvement of energy metabolism, up-regulation of anti-apoptotic Bcl-2 protein and down-regulation of pro-apoptotic Bax protein, inhibition of cytochrome c release, reduction of caspase 9 and caspase 3 activity after oxidative stress and reduction of apoptotic cell death [8]–[14]. However, evidence how GBE influences the mitochondrial oxidative phosphorylation system (OXPHOS) in neuronal cells is lacking.

AD is characterized by amyloid-beta (Aβ)-containing plaques, neurofibrillary tangles, as well as synapse and neuron loss. Aβ which represents with tau the main neuropathological hallmarks of AD is supposed to play a pivotal role in the pathogenesis of the disease. Within the Aβ toxicity cascade, mitochondrial dysfunction and energy metabolism deficiencies have been recognized as earliest events [10], [15] and have been correlated with impairments of cognitive abilities in AD clinical scenario [16]. The most consistent defect in mitochondrial electron transport system (ETS) enzymes in AD is the deficiency in cytochrome c oxidase (complex IV) activity in post-mortem brain tissues of AD patients [17]–[19] and APP transgenic mice [20], as well as in other tissues, such as platelets from AD patients and AD cybrid cells [21], [22]. Moreover, abnormal mitochondrial dynamics may play a role in mitochondrial dysfunction in AD [23], [24]. Interestingly, few years ago, the target of interest of Aβ toxic species switched from its extracellular, fibrillar form to its soluble, oligomeric form [23], [25] emphasising the important early role of mitochondria in AD pathogenic pathways [10], [24], [26], [27].

In line with these findings, we recently reported that chronic exposure to Aβ in human neuroblastoma cells (SH-SY5Y) over-expressing human wild-type APP (APP cells) resulted in an impairment of the respiratory capacity of OXPHOS and a drop in ATP generation by complex V which in turn may initiate cell death pathway [28].

Therefore, the aim of the present study was to investigate the potential effect of standardized GBE LI 1370 on Aβ-induced mitochondrial dysfunction in APP cells. In addition to the measurement of oxygen consumption in whole cells as well as of isolated mitochondria, we examined activities of mitochondrial enzymes assembling the ETS. Based on previous studies [11], [29], [30] we decided to focus on the more important mitochondrial respiratory enzymes implicated in AD and aging, that are the complexes I, III and IV. Furthermore, we determined ATP and reactive oxygen species (ROS) levels as well as the mitochondrial membrane mass. Finally, we studied the mitochondrial DNA/nuclear DNA ratio using real-time PCR to determine changes at the genetic level.

## Results

### GBE did not modify cells morphology but decreased oxidative stress

**Figure 1:**
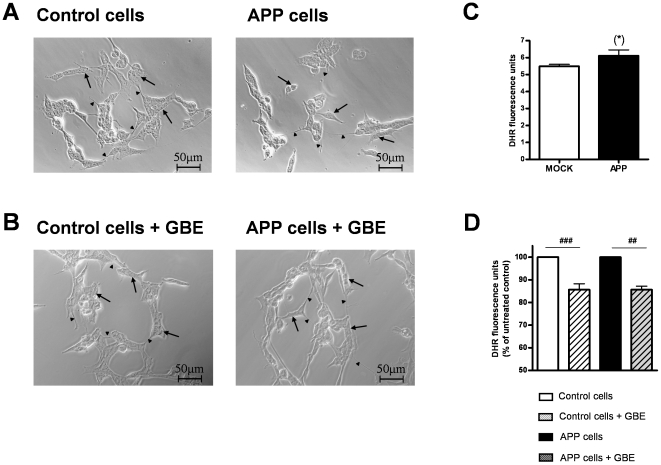
GBE did not modify cell morphology but decreased reactive oxygen species (ROS) levels. Morphological analysis of vector control and APP cells showed neuroblast-like morphology with differentiated perikaria (*arrows*) and occasional short neurites (*arrowheads*). **A**) Transfection of SH-SY5Y cells with cDNAs (pCEP4 vector) containing the vector alone or the entire coding region of wild-type human APP (APP cells) and **B**) GBE treatment (100 µg/ml; 24 h) did not significantly change general cell morphology. Scale bars: 50 µm. At least 200 cells were analyzed for each cell line and condition. **C**) Mitochondria-associated ROS levels measured after incubation with DHR (DHR fluorescence units/1×10^5^ cells). ROS levels showed a trend to be increased in APP cells compared to control cells (^(^*^)^p = 0.082, unpaired student's t-test, n = 11, values represent the means ± S.E.). **D**) ROS levels (DHR fluorescence units/1×10^5^ cells normalized to the respective untreated control and APP cells = 100%) were significantly decreased after treatment with GBE (100 µg/ml; 24 h). Values represent the means ± S.E., number of pairs n = 5, paired student's t-test: ##, p<0.01; ###, p<0.001 GBE treated versus corresponding untreated cells.

As described in our previous study [28], transfection of SH-SY5Y cells with cDNAs (pCEP4 vector) containing the vector alone or the entire coding region of human APP (APP695) did not significantly change the general morphological aspect of cells (Fig. 1A). Similarly, vector control as well as APP cells conserved their neuroblast-like morphology with differentiated perikaria and short neurites after treatment with GBE (Fig. 1B). APP cells showed a slight trend to increased ROS levels compared to controls cells (Fig. 1C). Mitochondria-associated ROS levels decreased significantly in both cell lines after treatment with GBE (100 µg/ml) for 24 h (Fig. 1D).

### GBE ameliorated OXPHOS capacity and restored Aβ-induced deficits

**Figure 2:**
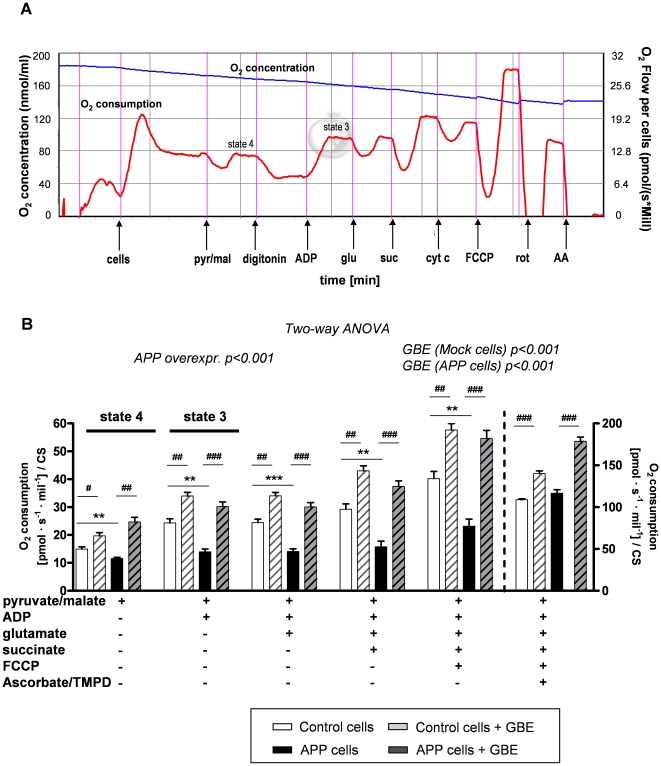
GBE treatment improved mitochondrial capacity. **A**) Representative diagram of measurement protocol of oxygen (O_2_) consumption in whole cells. O_2_ flux and O_2_ consumption by vital cells were measured after addition of different agents: pyruvate/glutamate (*pyr/glu*), digitonin (*dig*), ADP, glutamate (*glu*), succinate (*suc*), cytochrome c (*cyt c*), FCCP, rotenone (*rot*), antimycine A (*AA*). **B**) Two-way ANOVA revealed a significant Aβ-induced decrease of the total cellular respiration in APP cells compared to that of control cells (group effect: control vs. APP p<0.001). Two-way ANOVA revealed also a significant effect of GBE (100 µg/ml; 24 h) on total O_2_ consumption. Indeed, respiratory rates of mitochondria were increased in GBE-treated control and APP cells corresponding to that of their respective cell type (Two-way ANOVA, effect of GBE treatment in control cells p<0.001 and APP cells p<0.001). Values represent the means ± S.E. from n = 5 assays (measurements of control and APP cells were performed in parallel). Post-hoc analysis for single experimental respiratory conditions: GBE treatment effect, paired student's t-test, number of pairs n = 5: #, p<0.05, ##, p<0.01; ###, p<0.001 GBE treated versus corresponding untreated cells; and effect of Aβ, unpaired student's t-test, n = 5, **, p<0.01; ***, p<0.001 APP versus control cells). Values represent means ± S.E.

To investigate the protective effect of GBE against Aβ toxicity at the mitochondrial level, we used a high-resolution respiratory protocol established lately by our group [28]. Thus, physiological substrate combinations (pyruvate, glutamate and malate as substrates for complex I and succinate for complex II) were used to obtain mitochondrial bioenergetics approaching the most of physiological states in whole cells (Fig. 2A). We compared OXPHOS that is the whole ETS composed of the four mitochondrial enzymes (complex I to complex IV) and the F_1_F_0_ATP synthase of vital control and APP cells after pre-treatment with GBE for 24 h. As already previously observed [28], APP cells exhibited a significant impairment of OXPHOS capacity (Fig. 2B). Of note, GBE was able to significantly ameliorate the global failure of mitochondrial respiration in APP cells and increased oxygen consumption in control cells as well (Fig. 2B). Importantly, GBE-treated control and APP cells presented comparable mitochondrial respiratory rate validating the protective role of GBE on stabilization and normalization of mitochondrial capacity, respectively (Fig. 2B).

**Figure 3:**
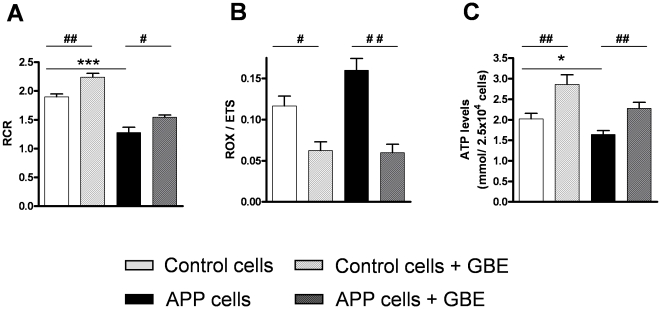
GBE modulated mitochondrial flux control ratios and rose ATP synthesis. **A**) Respiratory control ratio (RCR) represents the mitochondrial coupling state. RCR was decreased in APP cells and increased in GBE-treated control and APP cells. **B**) ROX/ETS yields an index of the magnitude of residual oxygen consumption relative to the maximum oxygen consumption capacity. This ratio was decreased in GBE-treated control and APP cells. **C**) ATP levels were decreased in APP cells and increased in GBE-treated control and APP cells. (**A–C**) Values represent the means ± S.E., GBE treatment effect, paired student's t-test, number of pairs n = 5: #, p<0.05, ##, p<0.01 GBE treated versus corresponding untreated cells; and effect of Aβ, unpaired student's t-test, n = 5, *, p<0.05; ***, p<0.001 APP versus control cells.

To analyse the impact of GBE on metabolic states of mitochondrial respiration, two flux control ratios have been evaluated. First, the respiratory control ratio (RCR) which is an indicator of the coupling state of mitochondria was determined. State 3 is the rate of phosphorylating respiration in the presence of exogenous ADP, when mitochondria are actively making ATP, whereas state 4 is the rate of resting respiration, when all ADP has been consumed. State 4 is associated with proton leakage across the inner mitochondrial membrane, when mitochondria exhibit basal activity, i.e. they are respiring but not making ATP. Therefore, RCR represents the ADP-activated flux to measure coupled OXPHOS capacity (state3) divided by leak flux (state4). Whereas increased Aβ levels led to a decrease of RCR in APP cells, treatment with GBE significantly increased the ratio in both cell types (Fig. 3A). Secondly, ROX/ETS yields an index of the magnitude of residual oxygen consumption relative to maximum oxygen consumption capacity. This ratio was decreased in both cell types after treatment with GBE with the strongest effect in APP cells (Fig. 3B). Both flux control ratio changes indicate an increase of the coupling state of mitochondria leading to a better efficiency of OXPHOS. Consistent with this result, we observed a rise of ATP levels which are closely correlated with a higher complex V activity, the final OXPHOS enzyme, in GBE-treated control and APP cells (Fig. 3C).

Taken together, respiratory analyses showed that GBE enhanced metabolic pathways by increasing the coupling state of OXPHOS promoting finally a rise of ATP synthesis in both cell types but with the strongest effects on APP cells. As a consequence, GBE-treated control and APP cells presented comparable mitochondrial respiratory system capacity suggesting a complete restoration of Aβ-induced deficits in energy metabolism.

### GBE improved oxygen consumption in isolated mitochondria from APP cells and led to an up-regulation of mitochondrial DNA

**Figure 4:**
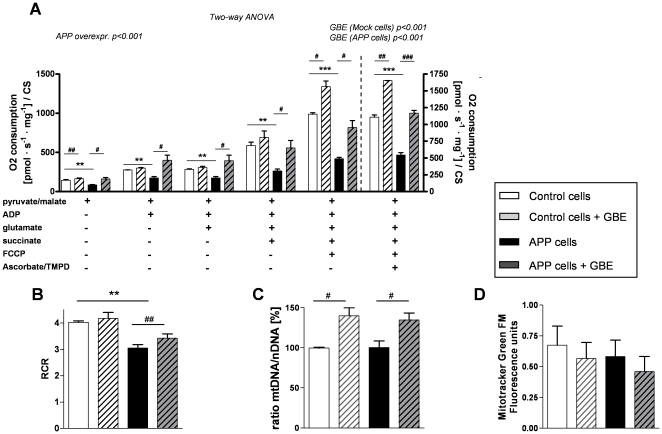
GBE enhanced oxygen consumption of isolated mitochondria and led to an up-regulation of mitochondrial DNA. **A**) Two-way ANOVA revealed a significant Aβ-induced decrease of the total cellular respiration in isolated mitochondria from APP cells compared to that of control cells (group effect: control vs. APP p<0.001). Two-way ANOVA revealed also a significant effect of GBE (100 µg/ml; 24 h) on total O_2_ consumption of isolated mitochondria. Respiratory rates of mitochondria were increased in mitochondria from GBE-treated control and APP cells corresponding to that of their respective untreated cell type (Two-way ANOVA, effect of GBE treatment in control cells p<0.001 and APP cells p<0.001). Values represent the means ± S.E. from n = 3 assays (measurements of control and APP cells were performed in parallel). Post-hoc analysis for single experimental respiratory conditions: GBE treatment effect, paired student's t-test, number of pairs n = 3: #, p<0.05, ##, p<0.01; ###, p<0.001 GBE treated versus corresponding untreated cells; and effect of Aβ, unpaired student's t-test, n = 3, **, p<0.01; ***, p<0.001 APP versus control cells). **B**) RCR of isolated mitochondria was decreased in APP cells (effect of Aβ, unpaired student's t-test, n = 3, **, p<0.01, APP versus control cells) and increased in GBE-treated APP cells (GBE treatment effect, paired student's t-test, number of pairs n = 3: ##, p<0.01, GBE treated versus untreated APP cells). **C**) RT-PCR analyses revealed an increase of the mitochondrial DNA/nuclear DNA ratio from GBE-treated control and APP cells (100 µg/ml; 24 h) compared to their corresponding untreated cells (number of pairs n = 5, #, p<0.05). **D**) Using MitoTracker Green FM (fluorescence units/1×10^5^ cells), no change in mitochondrial mass was observed. (**A–D**) Values represent means ± S.E.

To confirm the improvement of mitochondrial performance observed in whole cells, we investigated oxygen consumption of mitochondria that were isolated from control and APP cells after 24 h treatment with GBE. Consistent with our results on whole cells, isolated mitochondria from APP cells still exhibited a significant impairment of OXPHOS capacity and reduced RCR compared to mitochondria isolated from control cells (Fig. 4A and B). Again, GBE-induced enhancement of oxygen consumption was significantly present in both cell lines (Fig. 4A) with the strongest improvement in APP cells (Fig. 4A and B) suggesting a regulatory effect of GBE at the mitochondrial level even after its removal. Of note, this functional enhancement can be associated with an increase of the mitochondrial DNA/nuclear DNA ratio after treatment with GBE (Fig. 4C) but without a change of the mitochondrial membrane mass (Fig. 4D).

### GBE modulated the activity of respiratory complexes

**Figure 5:**
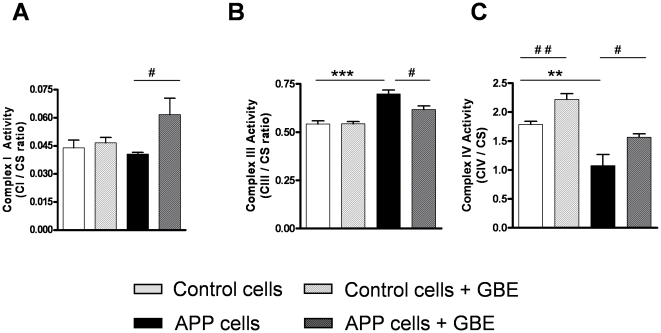
GBE modulated the activities of mitochondrial ETS enzymes. **A**) Complex I activity (CI/CS ratio) was increased in GBE-treated (100 µg/ml; 24 h) APP cells. **B**) Complex III activity (CIII/CS ratio) was increased in APP cells and decreased in GBE-treated APP cells (100 µg/ml; 24 h). **C**) Complex IV activity (CIV/CS ratio) was decreased in APP cells and increased in GBE-treated control and APP cells (100 µg/ml; 24 h). (**A–C**) Values represent the means ± S.E., GBE treatment effect, paired student's t-test, number of pairs n = 4–6: #, p<0.05, ##, p<0.01 GBE treated versus corresponding untreated cells; and effect of Aβ, unpaired student's t-test, n = 4–6, **, p<0.01; ***, p<0.001 APP versus control cells.

To improve the mitochondrial respiratory capacity, GBE may act on one or several mitochondrial enzymes. To study this hypothesis, activities of individual respiratory complexes have been investigated. Complex I activity significantly increased after GBE treatment selectively in APP cells (Fig. 5A), whereas complex II activity was unaffected by GBE in both cell lines (data not shown). Complex III activity which was markedly increased in APP cells was normalized to the level of control cells after treatment with GBE (Fig. 5B). Complex IV activity which was decreased in APP cells, significantly increased in control and APP cells after treatment with GBE (Fig. 5C).

### GBE partially decreased Aβ secretion of APP cells

**Figure 6:**
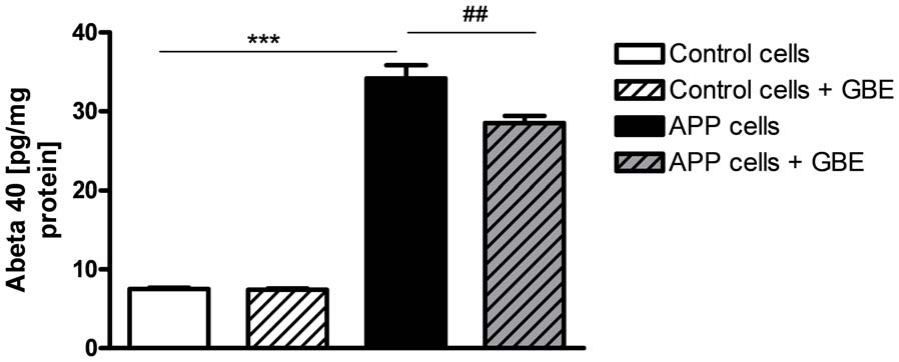
GBE decreased secretion of Aβ1-40 in APP cells. Stably transfected APP cells secreted significantly higher amounts of Aβ1-40 into the medium than control cells bearing the empty vector. Secreted Aβ1-40 was significantly decreased in GBE-treated APP cells (100 µg/ml; 24 h). Values represent the means ± S.E., GBE treatment effect, paired student's t-test, number of pairs n = 9: ##, p<0.01; GBE treated versus untreated APP cells, and effect of APP overexpression, unpaired student's t-test, n = 11–13: ***, p<0.001 APP versus control cells.

To delineate the relationship between Aβ level and GBE, we further examined the levels of secreted Aβ1-40 in control and APP cells. Figure 6 shows that APP cells secreted Aβ to a 3–4 fold higher extent than control cells bearing the empty vector. Of note, treatment with GBE significantly decreased medium Aβ levels in APP cells of about 20% (medium Aβ1-40, APP cells: 100%±4.8%, APP cells + GBE: 83.22%±2.6%, control cells: 22.03 ±0.4%, control cells + GBE: 21.06%±0.5%).

## Discussion

In this study, we present for the first time clear evidence that under physiological conditions GBE improved metabolic energy pathways by increasing the coupling state of mitochondria. Importantly, the comparison of the mitochondrial energetic capacity in both cell types after treatment with GBE indicates a recovery of the disturbed bioenergetic homeostasis found in APP cells. Notably, GBE-enhancing effect on OXPHOS was still present in mitochondria after their removal from cells suggesting possible continuing, regulatory actions of GBE at the mitochondrial level. These results corroborate findings showing an increase of the coupling state of isolated mitochondria by GBE in ischemia models [31] and findings demonstrating that GBE ameliorated oxygen consumption of mitochondria from rat heart mainly driven by effects of GBE on complex I and III [29]. The capacity of mitochondria to re-phosphorylate ADP in state 3 is positively dependent on the degree of coupling. In accordance with this assumption, we also observed an enhancement of ATP levels in both cell types after treatment with GBE. Moreover, the respiration rate in state 3 is determined by the activity of mitochondrial enzymes of the whole ETS and ATP-synthase [29], [30]. Thus, the global improvement of mitochondria functionality may be explained by the action of GBE on one or several mitochondrial enzymes. The increased activity of complex III in APP cells that can be interpreted as a compensatory mechanism to rescue Aβ-induced mitochondrial defects [29] normalized after treatment with GBE. This effect can be taken as a back to a physiological functionality, i.e. the over-activity of complex III in untreated APP cells was down-regulated in the presence of GBE which in turn up-regulated complex IV activity and ATP synthesis, both markedly reduced in our AD cell model. Surprisingly, complex I activity was increased only in APP cells after treatment with GBE while it was unchanged in control cells. APP cells did not exhibit an Aβ-induced defect in complex I activity compared to control cells [29]. GBE by increasing the coupling mitochondrial state may improve complex I activity. Complex I as a main ROS producer may also be a key element in GBE efficacy. However, this can not explain why the GBE effect on complex I was only found in APP cells. One can speculate that a damaged ETS with changes in downstream complexes, such as complex III and IV in APP cells, might force the emergence of this effect. Our study is further in line with recent findings studying the effect of GBE after *in vivo* treatment on mitochondrial membrane potential in dissociated brain cells from aged mice after inhibiting OXPHOS using specific inhibitors of complexes [11]. Here, GBE showed protective effects on mitochondrial membrane potential at the complexes I, IV and V [11]. Even if the exact molecular mechanisms by which GBE stabilizes mitochondrial enzymes is not yet clear, it can be speculated that besides other regulatory effects on mitochondrial components: (i) GBE may act as an antioxidant scavenging superoxide anion generated by electron leakage which occurs mainly at complexes I and III. (ii) GBE may also scavenge nitric oxide leading to an increase of complex IV activity which is the main target of NO [32]. Consequently, the protection of mitochondrial complexes by GBE may not only lead to an increase of mitochondrial performance (coupling state) but also to a decrease of ROS production. Moreover, some authors proposed that GBE provides anti-apoptotic effects [8], [9], [33]. Thus, GBE significantly attenuated mitochondria-initiated apoptosis and decreased the activity of caspase 3, a key enzyme in the apoptosis cell signalling cascade, in neuroblastoma cells stably expressing an AD-associated double mutation in APP [12]. In addition, GBE inhibited formation of Aβ-fibrils [12] either by a direct interaction with Aβ [34], [35] or by modulation of its precursor APP [36] and GBE flavonols were able to reduce Aβ levels in vitro and in the brain from transgenic Alzheimer mice [37]. We also observed that GBE treatment partially decreased Aβ generation in APP cells. Therefore, it might be possible that GBE restored impaired OXPHOS pathway through decreasing Aβ level, since Aβ interacts with mitochondria [27], [29], [31], [38], [39] and Aβ levels determine the extent of mitochondrial dysfunction [38]. Considerable evidence exists that Aβ can increase the production of ROS [40]. Consistent, we found a slight increase in ROS levels in APP cells compared to control cells suggesting that cellular antioxidant defences are still able to counterbalance excessive ROS production at least in part in our cell culture model.

Of note, we observed an up-regulation of mitochondrial DNA in cells treated with GBE, suggesting that GBE may act not only at a functional level of mitochondria but also at their genetic one. The number of mitochondrial DNA copies per mitochondrion as well as mitochondria per cell is exquisitely calibrated on cellular energy demand. Mitochondrial DNA (mtDNA) encodes important subunits of catalytic cores of mitochondrial respiratory complexes constituting OXPHOS. Consequently, in our study the increase of mtDNA associated with the increase of OXPHOS performance (coupling state) may facilitate the assembly of mitochondrial complexes into supercomplexes to optimize the respiratory activity. This idea is in agreement with the results of D'Aurelio and coworkers who revealed a correlation of improved mitochondrial respiration with the presence of supercomplexes [41]. Additionally, Tendi and colleagues [33] showed that GBE up-regulated mitochondrial gene expression of a mtDNA encoded subunit of complex I (ND1) in PC12 cells which may perfectly correspond to the increase of complex I activity detected in our GBE-treated APP cells. Moreover, the transcript level of the anti-apoptotic Bcl-2-like protein was found to be elevated, whereas the transcript level of the pro-apoptotic caspase 12 was decreased in PC12 cells after treatment with GBE [9], [12]. Although further studies are necessary, we can speculate that a GBE treatment may not only enhance mtDNA as an early event after acute treatment, but may instigate mitochondria biogenesis as a long-term effect after chronic exposure [42]. In line with this idea, recent studies showed that GBE induced hippocampal neurogenesis, a very energy demanding process, in young and old transgenic AD mouse model (TgAPP/PS1) after receiving a diet supplemented with GBE for 1 month [43].

Oxidative stress and especially mitochondria-derived oxidative stress is thought to have an important role in the pathogenesis of AD, a disease that likely begins years, if not decades, before clinical onset of dementia. In line with this hypothesis, experimental data support the notion that antioxidants may prevent or cure pathological conditions of AD. With regard to beneficial effects of antioxidants on OXPHOS performance, only few data are available for other antioxidants than GBE. Thus, one study provided evidence that vitamin E which is considered the major lipophilic antioxidant in the human body has GBE-like beneficial effects at high doses (corresponding to a human daily intake of 1,300 mg vitamin E/day) on age-dependent decline of OXPHOS and enzyme activities I as well as IV in aging male mice brain [44]. But concerns have been raised about the long-term safety of vitamin E supplementation, particularly in such high doses, and no evidence is provided demonstrating similar effects of vitamin E on OXPHOS at lower and rather physiologically relevant doses. MitoQ10 (mitochondria-targeted coenzyme Q) has also antioxidant properties but only when electron flow within the respiratory chain or complex I proton pumping is already retarded [45], while, in contrast, MitoQ10 action in the intact respiratory chain led to increased ROS production [45]. This is also contrary to our results of GBE on OXPHOS performance and ROS levels under non-pathological conditions found in control cells. Several meaningful differences distinguish the mode of action of GBE from those other antioxidants. Due to different constituents in the extract such as flavonols, bilobalide, and ginkgolides, GBE seems to exhibit a unique spectrum of action probably via complementary effects of its constituents [11] on mitochondrial function including (i) direct radical scavenging properties, (ii) direct stabilization of mitochondria, (iii) indirect protection of mitochondria, and (iiii) up-regulation of mtDNA, leading to improved OXPHOS performance and energy homeostasis. Whereas a huge body of evidence from preclinical research support the notion that antioxidants protect against neurodegeneration, previous observational studies of antioxidants, including vitamin E and GBE, and dementia risk have yielded inconsistent results based on short- or long-term follow-up periods [7], [46] raising the question of the “window of opportunity” for successful prevention. Further studies should continue to assess points at which treatment might be most relevant to dementia risk [47].

In total, our findings and findings of others indicate substantial mitochondria modulating properties of GBE. We could clearly show that GBE improved OXPHOS performance and was able to restore Aβ-induced mitochondria failure. Hereby, the increase in mitochondrial content might represent an important early event. Taking into account the increasing interest in mitochondrial stabilization as intervention strategy in AD [48], the regulating mechanisms of GBE on mitochondria function suggested by this study qualifies this drug as therapeutic candidate.

## Materials and Methods

### Cell culture and treatment with GBE

In the present study, human neuroblastoma SH-SY5Y cells stably expressing vector alone (pCEP4, control cells) or the entire coding region of human wild-type APP (APP695, APP cells) were used as described previously [49]. APP cells secreted Aβ levels within pg/mL range (around 150 pg/mL and 35 pg/mg protein respectively Aβ1-40 compared to approximately 50 pg/mL and 10 pg/mg protein respectively Aβ1-40 secreted by vector control cells) [28]. Stably transfected cell clones were selected with hygromycin [49]. Cells were grown at 37°C in DMEM medium supplemented with 10% calf serum, 2 mM L-glutamine, and 0.3 µg/mL hygromycin. Cells were pre-treated with standardized Ginkgo biloba extract LI 1370 (Vifor SA, Switzerland) for 24 h before analyzing the cells. A dose response for GBE was tested by treating the cells with different GBE concentrations starting from 0.001 mg/ml up to 0.5 mg/ml in distinct assays. The optimum concentration of GBE yielding maximum beneficial effects (with regard to reduction of ROS levels, enhancement of RCR, and increase in ATP levels) in APP cells was 100 µg/ml (see figures S1, S2, and S3, respectively), but significant beneficial effects could be detected at concentration as low as 10 µg/ml (see ATP results, figure S3) and 50 µg/ml (see ROS and ATP results, figures S1 and S3). A similar dose response was found for control cells (see figures S1, S2, and S3, respectively). Based on these findings and in agreement with other in vitro studies [11], [50], a standard concentration of 100 µg/ml of GBE was used in the following assays. In further pre-experiments, we excluded effects of the vehicle.

### Phase contrast microscopy and morphological analysis

For the morphological analysis, cells were seeded at a density of 4×10^4^ cells/mL on coverslips previously coated with 0.05 mg/mL collagen. Phase contrast pictures were taken from living neuroblastoma cells using a Zeiss Axiolab microscope with a 40×/1,2 W Korr objective equipped with a digital camera Zeiss AxioCam MRc.

### Preparation of isolated mitochondria for the determination of the activities of the single complexes I-IV

Cells were incubated for 15 min in an ice-cold lysis buffer (75 mM NaCl, 1 mM NaH_2_PO_4_, 8 mM Na_2_HPO_4_, 250 mM sucrose, 1 mM Pefabloc, 0.05% digitonine, complete protease inhibitor mixture tablets® (Roche Diagnostics)). Then, cells were homogenized with a glass homogenizer (10 strokes at 400 rpm and 5 strokes at 700 rpm), and the resulting homogenate was centrifuged at 800 *g* for 10 min at 4°C to remove nuclei and tissue particles. The supernatant 1 (S1) was saved and the pellet resuspended in the lysis buffer. The homogenization step as well as the low-speed centrifugation step was repeated. The supernatant 2 (S2) was saved and added to S1. Combined mitochondria-enriched supernatants (S1+S2) were centrifuged at 20,000 *g* for 15 min at 4°C to obtain the mitochondrial fraction. The mitochondrial pellet was resuspended in PBS and stored at 4°C until use, followed by determination of protein content [51].

### Complex I activity

A total of 240 µg of isolated mitochondria was solubilized in n-dodecyl β-D-maltoside (20%). NADH-ubiquinone oxidoreductase activity was measured at 30°C in a buffer containing 2 mM Na^+^/MOPS, 50 mM NaCl, and 2 mM KCN, pH 7.2, using 100 µM *n*-decylubiquinone (DBQ) and 100 µM NADH as substrates and 5 µM rotenone as inhibitor [20], [28], [52], [53]. Oxidation rate of NADH were recorded with a Shimadzu Multi-Spec-1501 diode array spectrophotometer (ε_340–400 nm_ = 6.1 mM^−1^.cm^−1^). Complex I activity was normalized to citrate synthase activity to take into account variations in the amount of mitochondrial and non-mitochondrial protein contamination between the samples [28], [54] and was given as CI/CS ratio.

### Complex III activity

The oxidation of 50 µM decylubiquinol obtained by complex III was determined using cytochrome *c* as an electron acceptor as described previously [28], [55]. Briefly, decylubiquinol was prepared by dissolving decylubiquinone (10 mM) in ethanol acidified to pH 2. The quinone was reduced with excess solid sodium borohydride. Decylubiquinol was extracted into diethylether:cyclohexane (2∶1, v/v) and evaporated to dryness under nitrogen gas, dissolved in ethanol acidified to pH 2. The assay was carried out in a medium containing 35 mM KH_2_PO_4_, 5 mM MgCl_2_, 2 mM KCN (pH 7.2), supplemented with 2.5 mg/mL BSA, 15 µM cytochrome *c*, 0,6 mM n-dodecyl β-D-maltoside and 5 µg/mL rotenone. The reaction was started with 10 µg of mitochondrial protein and the enzyme activity was measured at 550 nm. The extinction coefficient used for cytochrome *c* was 18.5 mM^−1^.cm^−1^. Complex III activity was normalized to citrate synthase activity to take into account variations in the amount of mitochondrial and non-mitochondrial protein contamination between the samples [28], [54] and was given as CIII/CS ratio.

### Complex IV activity

Cytochrome *c* oxidase activity was determined in intact isolated mitochondria (100 µg) using the Cytochrome *c* Oxidase Assay Kit (Sigma-Aldrich GmbH, Buchs, Switzerland). The colorimetric assay was based on the observation that a decrease in absorbance at 550 nm of ferrocytochrome *c* was caused by its oxidation to ferricytochrome *c* by cytochrome *c* oxidase. The Cytochrome *c* Oxidase Assay was performed as described previously [28], [53], [56]. Complex IV activity was normalized to citrate synthase activity to take into account variations in the amount of mitochondrial and non-mitochondrial protein contamination between the samples [28], [54] and was given as CIV/CS ratio.

### Reactive oxygen species (ROS) levels

Cells were plated the day before at a density of 1×10^5^ cells/well in a 48 well plate. The mitochondria-associated ROS levels were determined using the fluorescent dye dihydrorhodamine (DHR) at a concentration of 10 µM. The cells were loaded for 15 min with the dye. After washing twice with HBSS, the fluorescence was determined with the Victor2 reader multiplate (PerkinElmer Life Sciences) at 485 nm (excitation)/535 nm (emission).

### Oxygen consumption of vital cells

Mitochondrial oxygen consumption was measured at 37°C using an Oroboros Oxygraph-2k system. In contrast to the respiratory protocol measuring oxygen consumption of isolated mitochondria in a rather artificial experimental environment, this protocol enables the determination of oxygen consumption in whole, vital cells under the most physiological conditions. Five millions of cells were added to 2 mL of a mitochondrial respiration medium containing 0.5 mM EGTA, 3 mM MgCl_2_, 60 mM K-lactobionate, 20 mM taurine, 10 mM KH_2_P0_4_, 20 mM HEPES, 110 mM sucrose, 1g/l BSA (pH 7.1). To measure state 4 ( =  state 2) of complex I, 5 mM pyruvate and 2 mM malate were added and cells were permeabilised with 15 µg/mL digitonin. Afterwards, 2 mM ADP was added to measure state 3, and in order to increase the respiratory capacity, 10 mM glutamate was added. To study the effect of convergent complex I+II electron input on respiration, 10 mM of succinate was added [50]. The integrity of mitochondrial membrane was checked through the addition of 10 µM cytochrome c. After determining coupled respiration, 0.4 µM FCCP (Carbonyl cyanide p-(trifluoro-methoxy) phenyl-hydrazone) was added and respiration was measured in the absence of a proton gradient. In order to inhibit complex I and III activities 0.5 µM rotenone and 2.5 µM antimycine A, respectively were added. Then, 2 mM ascorbate and 0.5 mM TMPD were added to have access to complex IV activity. Finally, 100 mM sodium azide was added to inhibit the mitochondrial respiration. Control and APP cells untreated or treated with GBE were measured in parallel pairs using the same conditions (crossover design).

### Oxygen consumption of isolated mitochondria

The respiratory protocol used for isolated mitochondria was the same than this for whole cells except for the following points: (i) isolated mitochondria (0.5 mg) were added to 2 mL of a mitochondrial respiration medium containing 65 mM sucrose, 10 mM potassium phosphate, 10 mM Tris-HCl, 10 mM MgSO_4_, and 2 mM EDTA (pH 7.0), (ii) digitonin was not added and (iii) 0.05 µM of FCCP was sufficient to obtain the maximal mitochondrial respiration [53].

### Determination of ATP levels

Cells were plated one day before at a density of 2.5×10^4^ cells/well in a white 96 well plate. The assay kit is based on the bioluminescent measurement of ATP (ViaLightTM HT, Cambrex Bio Science, Lonza, Rockland, USA). The bioluminescent method utilizes the enzyme luciferase, which catalyses the formation of light from ATP and luciferin. The emitted light was linearly related to ATP concentration and was measured using a luminometer [48], [53].

### Estimation of mitochondrial membrane mass

Cells were plated the day before at a density of 1×10^5^ cells/well in a 48 well plate. Mitochondrial membrane mass was measured using the cell-permeable mitochondria-selective dye MitoTracker Green FM (Molecular Probes, Invitrogen, Lucerne, Switzerland) (400 nM, 15 min). This probe accumulates in active mitochondria independently of mitochondrial membrane potential and then reacts with accessible thiol groups of proteins and peptides [57]. Fluorescence was determined using a Fluoroskan Ascent FL multiplate reader (Labsystems) at 490 nm (excitation) /516 nm (emission).

### Quantitative real-time PCR for determination of mitochondrial DNA/nuclear DNA ratio

Samples consisted of confluent grown SH-SY5Y cells (APP or vector, untreated or treated for 24 h with GBE 100 µg/ml). Total DNA was extracted with the Qiagen FlexiGen Kit (Qiagen, Basel, Switzerland) according to the manufacturer's protocol. To determine the mitochondrial DNA (mtDNA) content in relation to nuclear DNA (nDNA) for each sample [58], [59], [60] a standard curve was generated via extracted DNA from pooled SH-SY5Y cell populations. For this purpose, mitochondrial and genomic genes were amplified using the same primers that were used for the real-time PCR (see below) and templates were purified with the Qiagen QIAquick PCR Purification Kit (Qiagen, Basel, Switzerland). The absorption at 260 nm was measured to determine the gene copy number and produce serial standard dilutions.

Primers for the RT Q-PCR analysis of mitochondrial DNA (mtDNA)-tRNAleu [61] were mtF3212 (5′CACCAAGAACAGGGTTTGT3′) and mtR3319 (5′TGGCCATGGGTATGTT GTTAA3′) [62], those for nuclear DNA (nDNA), human polymerase γ accessory subunit gene (ASPOLG) were ASPG3F 5′GAGCTGTTGACGGAAAGGAG3′ and ASPG4R (5′CAG AAGAGAATCCCGGCTAAG3′) [63]. For each DNA extract, the nDNA and mtDNA gene respectively were quantified separately in triplicates and/or duplicates by real-time quantitative PCR in the presence of SYBR-green with the use of the Applied Biosystems StepOne™ cycler (Applied-Biosystems). PCR reactions contained 1x Power SYBR Green Master Mix (ABI P/N 4367659, Rotkreuz, Switzerland), 1 µM of each primer and 1 ng of DNA extract. The PCR amplification consisted of a single denaturation-enzyme-activation step of 10 min at 95°C, followed by 40cycles of 15 sec denaturation at 95°C and 60 sec of annealing/extension at 60°C. Fluorescence was measured at the end of each extension step. A standard curve of corresponding mtDNA and nDNA template equivalents with defined copy numbers were included in each run to quantify the content of mtDNA and nDNA in each sample. Data thus obtained were analyzed by using the cycle threshold (C_T_) of each amplification reaction relating it to its respective standard curve. Results from the quantitative PCR were expressed as the ratio of the mean mitochondrial DNA to the mean nuclear DNA content.

### Detection of Aβ levels

Detection of Aβ1-40 secretion was performed by using sandwich enzyme-linked immunosorbent assay (Invitrogen, Basel, Switzerland) according to the manufacturer's protocol. Secreted Aβ1-40 levels within the medium (Aβ1-40 pg/ml) were normalized to mg protein/ml. Values of intracellular Aβ1-40 as well as medium and intracellular Aβ1-42 levels were below detection limit and are therefore not shown.

### Statistical Analysis

Data were presented as mean ± S.E.M. For statistical comparisons, unpaired and paired Student's *t*-test, respectively, or Two-way ANOVA was used. P values less than 0.05 were considered statistically significant.

## Supporting Information

Figure S1 GBE decreased reactive oxygen species (ROS) levels in a dose response manner. Mitochondria-associated ROS levels measured after incubation with DHR (DHR fluorescence units/1×10^5^ cells normalized to the respective untreated control and APP cells). A) Control cells showed significantly decreased ROS levels after treatment with GBE (0.001–0.5 mg/ml; 24 h) significantly for concentrations between 0.05–0.5 mg/ml. B) GBE-treated APP cells (0.001–0.5 mg/ml; 24 h) exhibited significantly reduced ROS levels for the same concentration range 0.05–0.5 mg/ml compared to control cells. Values represent the means ± S.E., GBE treatment effect, paired student′s t-test, number of pairs n =  11: #, p<0.05, ##, p<0.01; GBE treated versus corresponding untreated control and APP cells.
(3.94 MB TIF)

Figure S2 GBE modulated mitochondrial flux control ratios in a dose response manner. Respiratory control ratio (RCR) was lower in APP cells than in control cells. After treatment with GBE (0.001–0.5 mg/ml; 24 h), RCR increased in control as well as in APP cells from 0.01 mg/ml up to 0.1 mg/ml GBE (maximum response in GBE treated APP cells). Values represent the means of 3 experiments.
(1.33 MB TIF)

Figure S3 GBE rose ATP synthesis in a dose response manner. A) Control cells showed increased ATP levels after treatment with GBE (0.001–0.5 mg/ml; 24 h) significantly for concentrations of 0.01 and 0.1 mg/ml. B) GBE-treated APP cells (0.001–0.5 mg/ml; 24 h) exhibited significantly increased ATP levels for concentrations ranged between 0.01–0.1 mg/ml. Values represent the means ± S.E., GBE treatment effect, paired student's t-test, number of pairs n =  6: #, p<0.05, ##, p<0.01; GBE treated versus corresponding untreated control and APP cells.
(0.74 MB TIF)

## References

[1] Kanowski S, Herrmann WM, Stephan K, Wierich W, Horr R (1996). Proof of efficacy of the ginkgo biloba special extract EGb 761 in outpatients suffering from mild to moderate primary degenerative dementia of the Alzheimer type or multi-infarct dementia.. Pharmacopsychiatry.

[2] Le Bars PL, Katz MM, Berman N, Itil TM, Freedman AM (1997). A placebo-controlled, double-blind, randomized trial of an extract of Ginkgo biloba for dementia. North American EGb Study Group.. JAMA.

[3] Napryeyenko O, Borzenko I (2007). Ginkgo biloba special extract in dementia with neuropsychiatric features. A randomised, placebo-controlled, double-blind clinical trial.. Arzneimittelforschung.

[4] Yancheva S, Ihl R, Nikolova G, Panayotov P, Schlaefke S (2009). Ginkgo biloba extract EGb 761(R), donepezil or both combined in the treatment of Alzheimer's disease with neuropsychiatric features: a randomised, double-blind, exploratory trial.. Aging Ment Health.

[5] Mix JA, Crews WD (2002). A double-blind, placebo-controlled, randomized trial of Ginkgo biloba extract EGb 761 in a sample of cognitively intact older adults: neuropsychological findings.. Hum Psychopharmacol.

[6] McCarney R, Fisher P, Iliffe S, van Haselen R, Griffin M (2008). Ginkgo biloba for mild to moderate dementia in a community setting: a pragmatic, randomised, parallel-group, double-blind, placebo-controlled trial.. Int J Geriatr Psychiatry.

[7] DeKosky ST, Williamson JD, Fitzpatrick AL, Kronmal RA, Ives DG (2008). Ginkgo biloba for prevention of dementia: a randomized controlled trial.. JAMA.

[8] Schindowski K, Leutner S, Kressmann S, Eckert A, Muller WE (2001). Age-related increase of oxidative stress-induced apoptosis in mice prevention by Ginkgo biloba extract (EGb761).. J Neural Transm.

[9] Smith JV, Burdick AJ, Golik P, Khan I, Wallace D (2002). Anti-apoptotic properties of Ginkgo biloba extract EGb 761 in differentiated PC12 cells.. Cell Mol Biol (Noisy-le-grand).

[10] Leuner K, Hauptmann S, Abdel-Kader R, Scherping I, Keil U (2007). Mitochondrial dysfunction: the first domino in brain aging and Alzheimer's disease?. Antioxid Redox Signal.

[11] Abdel-Kader R, Hauptmann S, Keil U, Scherping I, Leuner K (2007). Stabilization of mitochondrial function by Ginkgo biloba extract (EGb 761).. Pharmacol Res.

[12] Luo Y, Smith JV, Paramasivam V, Burdick A, Curry KJ (2002). Inhibition of amyloid-beta aggregation and caspase-3 activation by the Ginkgo biloba extract EGb761.. Proc Natl Acad Sci U S A.

[13] Eckert A, Keil U, Scherping I, Hauptmann S, Muller WE (2005). Stabilization of Mitochondrial Membrane Potential and Improvement of Neuronal Energy Metabolism by Ginkgo Biloba Extract EGb 761.. Ann N Y Acad Sci.

[14] Eckert A, Keil U, Kressmann S, Schindowski K, Leutner S (2003). Effects of EGb 761 Ginkgo biloba extract on mitochondrial function and oxidative stress.. Pharmacopsychiatry.

[15] Moreira PI, Carvalho C, Zhu X, Smith MA, Perry G (2010). Mitochondrial dysfunction is a trigger of Alzheimer's disease pathophysiology.. Biochim Biophys Acta.

[16] Carvalho C, Correia SC, Santos RX, Cardoso S, Moreira PI (2009). Role of mitochondrial-mediated signaling pathways in Alzheimer disease and hypoxia.. J Bioenerg Biomembr.

[17] Blass JP (2003). Cerebrometabolic abnormalities in Alzheimer's disease.. Neurol Res.

[18] Kish SJ, Bergeron C, Rajput A, Dozic S, Mastrogiacomo F (1992). Brain cytochrome oxidase in Alzheimer's disease.. J Neurochem.

[19] Valla J, Berndt JD, Gonzalez-Lima F (2001). Energy hypometabolism in posterior cingulate cortex of Alzheimer's patients: superficial laminar cytochrome oxidase associated with disease duration.. J Neurosci.

[20] Gibson GE, Sheu KF, Blass JP (1998). Abnormalities of mitochondrial enzymes in Alzheimer disease.. J Neural Transm.

[21] Hauptmann S, Scherping I, Drose S, Brandt U, Schulz KL (2009). Mitochondrial dysfunction: an early event in Alzheimer pathology accumulates with age in AD transgenic mice.. Neurobiol Aging.

[22] Cardoso SM, Proenca MT, Santos S, Santana I, Oliveira CR (2004). Cytochrome c oxidase is decreased in Alzheimer's disease platelets.. Neurobiol Aging.

[23] Wang X, Su B, Lee HG, Li X, Perry G (2009). Impaired balance of mitochondrial fission and fusion in Alzheimer's disease.. J Neurosci.

[24] Iijima-Ando K, Hearn SA, Shenton C, Gatt A, Zhao L (2009). Mitochondrial mislocalization underlies Abeta42-induced neuronal dysfunction in a Drosophila model of Alzheimer's disease.. PLoS One.

[25] Cardoso SM, Santana I, Swerdlow RH, Oliveira CR (2004). Mitochondria dysfunction of Alzheimer's disease cybrids enhances Abeta toxicity.. J Neurochem.

[26] Fernandez-Vizarra P, Fernandez AP, Castro-Blanco S, Serrano J, Bentura ML (2004). Intra- and extracellular Abeta and PHF in clinically evaluated cases of Alzheimer's disease.. Histol Histopathol.

[27] Lustbader JW, Cirilli M, Lin C, Xu HW, Takuma K (2004). ABAD directly links Abeta to mitochondrial toxicity in Alzheimer's disease.. Science.

[28] Rhein V, Eckert A (2007). Effects of Alzheimer's amyloid-beta and tau protein on mitochondrial function – role of glucose metabolism and insulin signalling.. Arch Physiol Biochem.

[29] Rhein V, Baysang G, Rao S, Meier F, Bonert A (2009). Amyloid-beta leads to impaired cellular respiration, energy production and mitochondrial electron chain complex activities in human neuroblastoma cells.. Cell Mol Neurobiol.

[30] Rhein V, Song X, Wiesner A, Ittner LM, Baysang G (2009). Amyloid-beta and tau synergistically impair the oxidative phosphorylation system in triple transgenic Alzheimer's disease mice.. Proc Natl Acad Sci U S A.

[31] Eckert A, Hauptmann S, Scherping I, Meinhardt J, Rhein V (2008). Oligomeric and fibrillar species of beta-amyloid (A beta 42) both impair mitochondrial function in P301L tau transgenic mice.. J Mol Med.

[32] Gnaiger E (2009). Capacity of oxidative phosphorylation in human skeletal muscle: new perspectives of mitochondrial physiology.. Int J Biochem Cell Biol.

[33] Janssens D, Michiels C, Delaive E, Eliaers F, Drieu K (1995). Protection of hypoxia-induced ATP decrease in endothelial cells by ginkgo biloba extract and bilobalide.. Biochem Pharmacol.

[34] Trumbeckaite S, Bernatoniene J, Majiene D, Jakstas V, Savickas A (2007). Effect of Ginkgo biloba extract on the rat heart mitochondrial function.. J Ethnopharmacol.

[35] Doussiere J, Ligeti E, Brandolin G, Vignais PV (1984). Control of oxidative phosphorylation in rat heart mitochondria. The role of the adenine nucleotide carrier.. Biochim Biophys Acta.

[36] Davey GP, Peuchen S, Clark JB (1998). Energy thresholds in brain mitochondria. Potential involvement in neurodegeneration.. J Biol Chem.

[37] Hou Y, Aboukhatwa MA, Lei DL, Manaye K, Khan I (2010). Anti-depressant natural flavonols modulate BDNF and beta amyloid in neurons and hippocampus of double TgAD mice.. Neuropharmacology.

[38] Keil U, Bonert A, Marques CA, Scherping I, Weyermann J (2004). Amyloid beta-induced changes in nitric oxide production and mitochondrial activity lead to apoptosis.. J Biol Chem.

[39] Eckert A, Schulz KL, Rhein V, Gotz J (2010). Convergence of amyloid-beta and tau pathologies on mitochondria in vivo.. Mol Neurobiol.

[40] Shi Q, Gibson GE (2007). Oxidative stress and transcriptional regulation in Alzheimer disease.. Alzheimer Dis Assoc Disord.

[41] Janssens D, Remacle J, Drieu K, Michiels C (1999). Protection of mitochondrial respiration activity by bilobalide.. Biochem Pharmacol.

[42] Cleeter MW, Cooper JM, Darley-Usmar VM, Moncada S, Schapira AH (1994). Reversible inhibition of cytochrome c oxidase, the terminal enzyme of the mitochondrial respiratory chain, by nitric oxide. Implications for neurodegenerative diseases.. FEBS Lett.

[43] Tendi EA, Bosetti F, Dasgupta SF, Stella AM, Drieu K (2002). Ginkgo biloba extracts EGb 761 and bilobalide increase NADH dehydrogenase mRNA level and mitochondrial respiratory control ratio in PC12 cells.. Neurochem Res.

[44] Navarro A, Gomez C, Sanchez-Pino MJ, Gonzalez H, Bandez MJ (2005). Vitamin E at high doses improves survival, neurological performance, and brain mitochondrial function in aging male mice.. Am J Physiol Regul Integr Comp Physiol.

[45] Plecita-Hlavata L, Jezek J, Jezek P (2009). Pro-oxidant mitochondrial matrix-targeted ubiquinone MitoQ10 acts as anti-oxidant at retarded electron transport or proton pumping within Complex I.. Int J Biochem Cell Biol.

[46] Laurin D, Masaki KH, Foley DJ, White LR, Launer LJ (2004). Midlife dietary intake of antioxidants and risk of late-life incident dementia: the Honolulu-Asia Aging Study.. Am J Epidemiol.

[47] Devore EE, Grodstein F, van Rooij FJ, Hofman A, Stampfer MJ (2010). Dietary antioxidants and long-term risk of dementia.. Arch Neurol.

[48] Moreira PI, Zhu X, Wang X, Lee HG, Nunomura A (2010). Mitochondria: a therapeutic target in neurodegeneration.. Biochim Biophys Acta.

[49] Bastianetto S, Ramassamy C, Dore S, Christen Y, Poirier J (2000). The Ginkgo biloba extract (EGb 761) protects hippocampal neurons against cell death induced by beta-amyloid.. Eur J Neurosci.

[50] Tchantchou F, Lacor PN, Cao Z, Lao L, Hou Y (2009). Stimulation of neurogenesis and synaptogenesis by bilobalide and quercetin via common final pathway in hippocampal neurons.. J Alzheimers Dis.

[51] Yao Z, Drieu K, Papadopoulos V (2001). The Ginkgo biloba extract EGb 761 rescues the PC12 neuronal cells from beta-amyloid-induced cell death by inhibiting the formation of beta-amyloid-derived diffusible neurotoxic ligands.. Brain Res.

[52] Colciaghi F, Borroni B, Zimmermann M, Bellone C, Longhi A (2004). Amyloid precursor protein metabolism is regulated toward alpha-secretase pathway by Ginkgo biloba extracts.. Neurobiol Dis.

[53] Augustin S, Rimbach G, Augustin K, Schliebs R, Wolffram S (2009). Effect of a short- and long-term treatment with Ginkgo biloba extract on amyloid precursor protein levels in a transgenic mouse model relevant to Alzheimer's disease.. Arch Biochem Biophys.

[54] D'Aurelio M, Gajewski CD, Lenaz G, Manfredi G (2006). Respiratory chain supercomplexes set the threshold for respiration defects in human mtDNA mutant cybrids.. Hum Mol Genet.

[55] Kuroiwa T, Ohta T, Kuroiwa H, Shigeyuki K (1994). Molecular and cellular mechanisms of mitochondrial nuclear division and mitochondriokinesis.. Microsc Res Tech.

[56] Tchantchou F, Xu Y, Wu Y, Christen Y, Luo Y (2007). EGb 761 enhances adult hippocampal neurogenesis and phosphorylation of CREB in transgenic mouse model of Alzheimer's disease.. FASEB J.

[57] Scheuermann S, Hambsch B, Hesse L, Stumm J, Schmidt C (2001). Homodimerization of amyloid precursor protein and its implication in the amyloidogenic pathway of Alzheimer's disease.. J Biol Chem.

[58] Bai RK, Wong LJ (2005). Simultaneous detection and quantification of mitochondrial DNA deletion(s), depletion, and over-replication in patients with mitochondrial disease.. J Mol Diagn.

[59] Bai RK, Perng CL, Hsu CH, Wong LJ (2004). Quantitative PCR analysis of mitochondrial DNA content in patients with mitochondrial disease.. Ann N Y Acad Sci.

[60] Cote HC, Brumme ZL, Craib KJ, Alexander CS, Wynhoven B (2002). Changes in mitochondrial DNA as a marker of nucleoside toxicity in HIV-infected patients.. N Engl J Med.

[61] Lowry OH, Rosebrough NJ, Farr AL, Randall RJ (1951). Protein measurement with the Folin phenol reagent.. J Biol Chem.

[62] Djafarzadeh R, Kerscher S, Zwicker K, Radermacher M, Lindahl M (2000). Biophysical and structural characterization of proton-translocating NADH-dehydrogenase (complex I) from the strictly aerobic yeast Yarrowia lipolytica.. Biochim Biophys Acta.

[63] David DC, Hauptmann S, Scherping I, Schuessel K, Keil U (2005). Proteomic and functional analyses reveal a mitochondrial dysfunction in P301L tau transgenic mice.. J Biol Chem.

