# Supplementary figures and images for: Ginkgo Biloba Extract Ameliorates Oxidative Phosphorylation Performance and Rescues Aβ-Induced Failure
Source: PLoS One. 2010 Aug 24;5(8):e12359. doi: 10.1371/journal.pone.0012359 (PMC2927422; doi:10.1371/journal.pone.0012359)

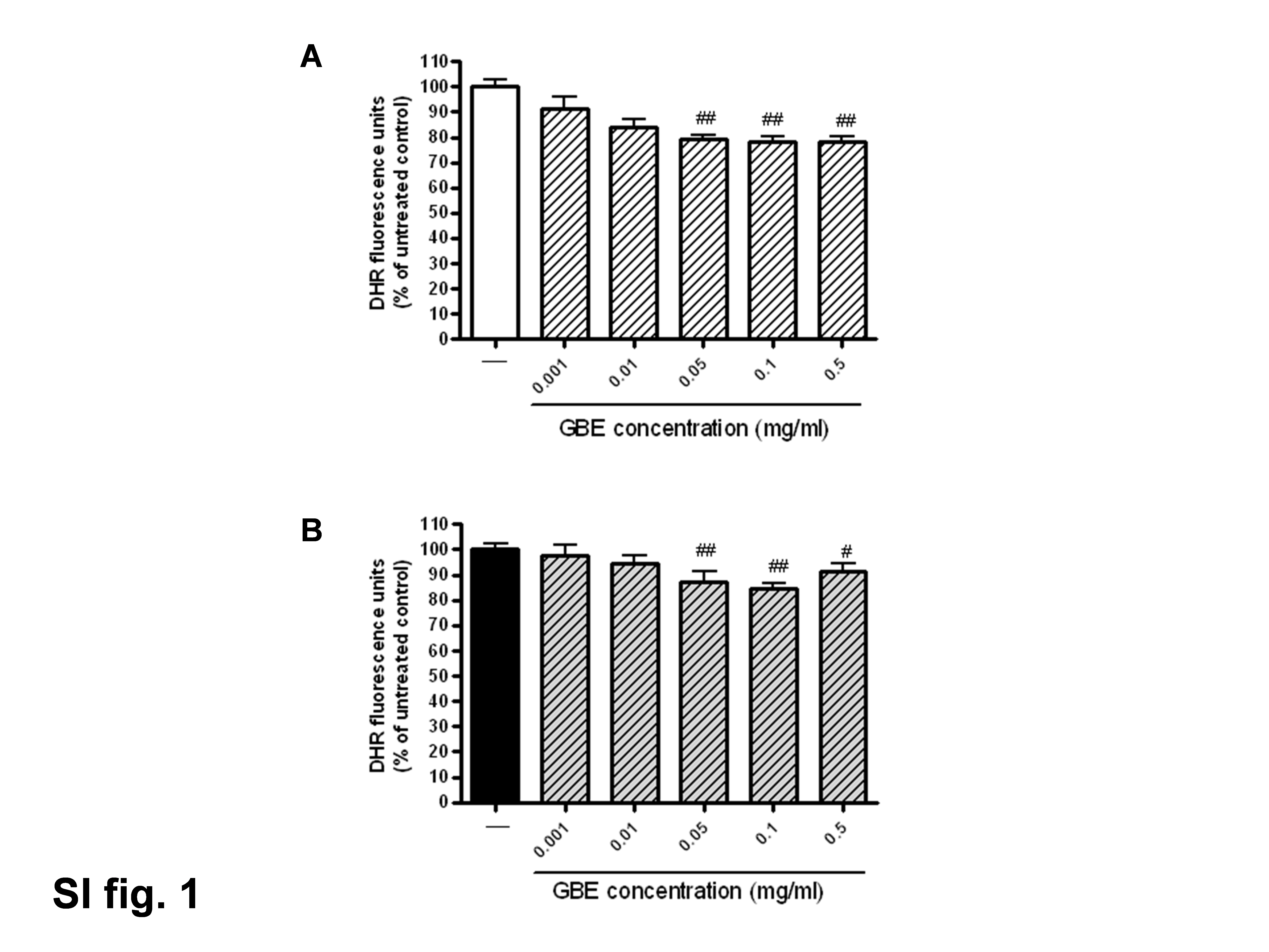

Supplement: Figure S1 — GBE decreased reactive oxygen species (ROS) levels in a dose response manner. Mitochondria-associated ROS levels measured after incubation with DHR (DHR fluorescence units/1×105 cells normalized to the respective untreated control and APP cells). A) Control cells showed significantly decreased ROS levels after treatment with GBE (0.001–0.5 mg/ml; 24 h) significantly for concentrations between 0.05–0.5 mg/ml. B) GBE-treated APP cells (0.001–0.5 mg/ml; 24 h) exhibited significantly reduced ROS levels for the same concentration range 0.05–0.5 mg/ml compared to control cells. Values represent the means ± S.E., GBE treatment effect, paired student′s t-test, number of pairs n = 11: #, p<0.05, ##, p<0.01; GBE treated versus corresponding untreated control and APP cells. (3.94 MB TIF) [file pone.0012359.s001.tif]

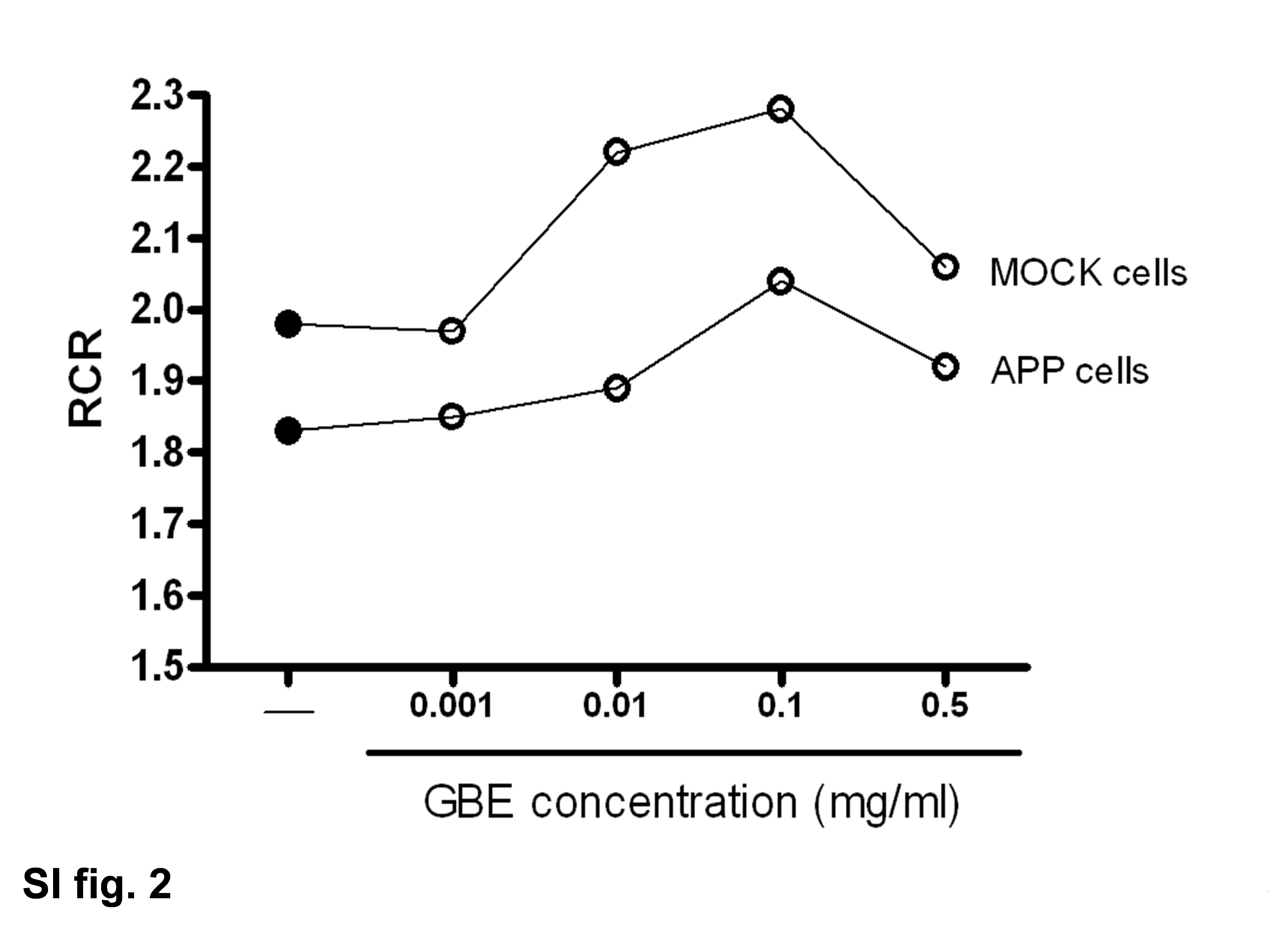

Supplement: Figure S2 — GBE modulated mitochondrial flux control ratios in a dose response manner. Respiratory control ratio (RCR) was lower in APP cells than in control cells. After treatment with GBE (0.001–0.5 mg/ml; 24 h), RCR increased in control as well as in APP cells from 0.01 mg/ml up to 0.1 mg/ml GBE (maximum response in GBE treated APP cells). Values represent the means of 3 experiments. (1.33 MB TIF) [file pone.0012359.s002.tif]

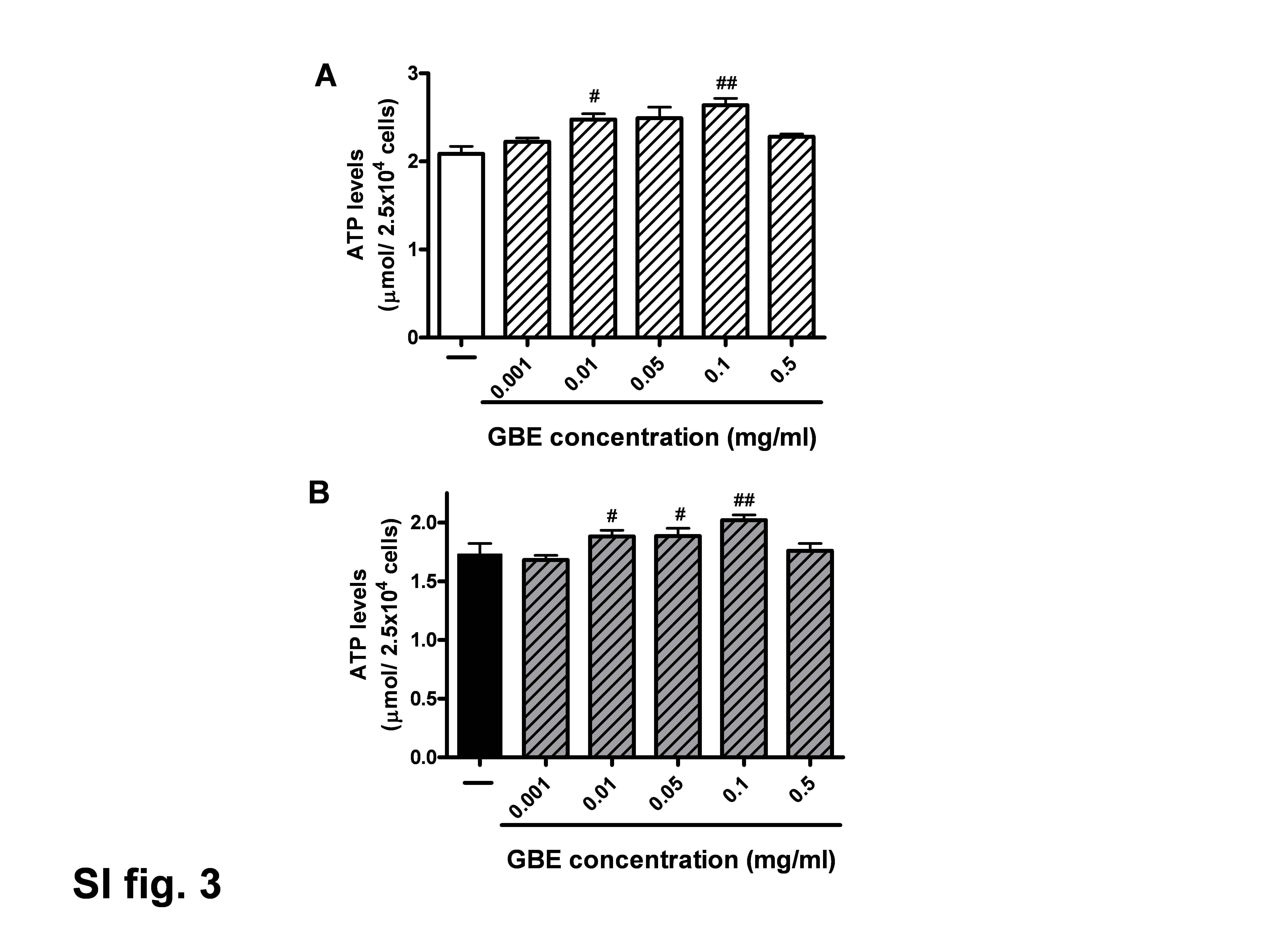

Supplement: Figure S3 — GBE rose ATP synthesis in a dose response manner. A) Control cells showed increased ATP levels after treatment with GBE (0.001–0.5 mg/ml; 24 h) significantly for concentrations of 0.01 and 0.1 mg/ml. B) GBE-treated APP cells (0.001–0.5 mg/ml; 24 h) exhibited significantly increased ATP levels for concentrations ranged between 0.01–0.1 mg/ml. Values represent the means ± S.E., GBE treatment effect, paired student's t-test, number of pairs n = 6: #, p<0.05, ##, p<0.01; GBE treated versus corresponding untreated control and APP cells. (0.74 MB TIF) [file pone.0012359.s003.tif]
